# A phase I study of an agonist anti-CD27 human antibody (CDX-1127) in patients with advanced hematologic malignancies or solid tumors

**DOI:** 10.1186/2051-1426-1-S1-P259

**Published:** 2013-11-07

**Authors:** Stephen Ansell, Donald Northfelt, Ian Flinn, Howard Burris, Shira Dinner, Victor Villalobos, Branimir Sikic, Lana Pilja, Michael Yellin, Tibor Keler, Thomas Davis

**Affiliations:** 1 Mayo Clinic, Rochester, MN, USA; 2 Mayo Clinic, Scottsdale, AZ, USA; 3 Sarah Cannon Research Institute, Nashville, TN, USA; 4 Stanford Cancer Institute, Stanford, CA, USA; 5 Celldex Therapeutics, Inc., Phillipsburg, NJ, USA

## 

CD27, a member of the tumor necrosis factor receptor superfamily, is constitutively expressed on the majority of mature T cells, memory B cells, and a subset of NK cells. We previously demonstrated anti-tumor and immunological properties of the fully human anti-CD27 mAb 1F5 (CDX-1127) in murine tumor models of hematologic and solid tumors. A Phase I dose escalation study of CDX-1127 in patients (pts) with recurrent or treatment-refractory B-cell malignancies and select solid tumors is ongoing. CDX-1127 (0.1 to 10 mg/kg IV) is administered as a single-dose with 28-day observation, followed by 4 weekly doses before restaging at Day 85. Up to 4 additional re-treatment cycles (consisting of 4 weekly doses) are permitted in pts with stable disease or tumor response. Dose-escalation in pts with solid tumors has been completed with preliminary results presented in a separate abstract. Flow cytometry and functional immune analysis of peripheral blood lymphocytes from these solid tumor pts showed an increase in expression of activation markers on T cells (HLA-DR), increases in NK cells, decrease in Tregs, and enhanced T cell response to various stimuli in in vitro assays. Tumor shrinkage was also seen. Dose-escalation in pts with hematologic malignancies is ongoing. To date, 13 B-cell lymphoma pts (3 Hodgkin, 3 follicular, 3 marginal zone, 4 diffuse large B-cell) have received 0.1, 0.3, or 1 mg/kg CDX-1127, with no DLT. Treatment-related toxicity, generally Grade 1-2, included fatigue, nausea, decreased appetite and anemia. One pt with Hodgkin lymphoma who had previously progressed following treatment with chemotherapy, autologous stem cell transplant, and brentuximab vedotin had a partial response (PR) with 77% shrinkage in measurable disease; treatment is ongoing. Three additional pts had stable disease (range: 4.5 to 11+ months), including a pt with follicular lymphoma who has completed 3 cycles of therapy and a pt with marginal zone lymphoma with -36% shrinkage in measurable disease. Flow cytometry and functional immune analysis of peripheral blood lymphocytes are being performed to assess the immunomodulatory activity of CDX-1127 in patients with hematological malignancies. Emerging results suggest that weekly dosing of CDX-1127 is well tolerated with promising biological and early clinical activity. In addition, these data position CDX-1127 well for combination therapy with conventional and immunotherapy approaches as demonstrated in preclinical models.

